# Major elective abdominal surgery acutely impairs lower limb muscle pyruvate dehydrogenase complex activity and mitochondrial function

**DOI:** 10.1016/j.clnu.2020.07.006

**Published:** 2021-03

**Authors:** Ryan Atkins, Dumitru Constantin-Teodosiu, Krishna K. Varadhan, Despina Constantin, Dileep N. Lobo, Paul L. Greenhaff

**Affiliations:** a MRC Versus Arthritis Centre for Musculoskeletal Ageing Research, School of Life Sciences, University of Nottingham, Queen's Medical Centre, Nottingham, NG7 2UH, UK; b Gastrointestinal Surgery, Nottingham Digestive Diseases Centre, University of Nottingham, Queen's Medical Centre, Nottingham, NG7 2UH, UK; c National Institute of Health Research (NIHR) Nottingham Biomedical Research Centre, Nottingham University Hospitals NHS Trust and University of Nottingham, Queen's Medical Centre, Nottingham, NG7 2UH, UK

**Keywords:** Abdominal surgery, Muscle inflammatory responses, Metabolic response, Muscle mitochondrial activity, Pyruvate dehydrogenase complex, ADP, adenosine diphosphate, ATP, adenosine triphosphate, ETC, electron transport chain, FOXO1, Forkhead box transcription factor 1, IL, interleukin, MAPR, maximal mitochondrial ATP production rates, MAFbx, muscle atrophy F-box protein, NADH, nicotinamide adenine dinucleotide, PDC, pyruvate dehydrogenase complex, PDK4, pyruvate dehydrogenase kinase isoform 4, TCA, tricarboxylic acid, TNF-α, tumour necrosis factor alpha, VL, vastus lateralis

## Abstract

**Background & aims:**

This *post hoc* study aimed to determine whether major elective abdominal surgery had any acute impact on mitochondrial pyruvate dehydrogenase complex (PDC) activity and maximal mitochondrial ATP production rates (MAPR) in a large muscle group (vastus lateralis -VL) distant to the site of surgical trauma.

**Methods:**

Fifteen patients undergoing major elective open abdominal surgery were studied. Muscle biopsies were obtained after the induction of anesthesia from the VL immediately before and after surgery for the determination of PDC and maximal MAPR (utilizing a variety of energy substrates).

**Results:**

Muscle PDC activity was reduced by >50% at the end of surgery compared with pre-surgery (p < 0.05). Muscle MAPR were comprehensively suppressed by surgery for the substrate combinations: glutamate + succinate; glutamate + malate; palmitoylcarnitine + malate; and pyruvate + malate (all p < 0.05), and could not be explained by a lower mitochondrial yield.

**Conclusions:**

PDC activity and mitochondrial ATP production capacity were acutely impaired in muscle distant to the site of surgical trauma. In keeping with the limited data available, we surmise these events resulted from the general anesthesia procedures employed and the surgery related trauma. These findings further the understanding of the acute dysregulation of mitochondrial function in muscle distant to the site of major surgical trauma in patients, and point to the combination of general anesthesia and trauma related inflammation as being drivers of muscle metabolic insult that warrants further investigation.

**Clinical trial registration:**

Registered at (NCT01134809).

## 1 Introduction

Whole-body insulin resistance accompanies surgical trauma, and appears to be proportional to the magnitude of injury incurred [1]. Skeletal muscle is the primary site for insulin-dependent glucose uptake, and surgical trauma appears to play an important role in the downregulation of muscle glucose uptake and oxidation following major abdominal surgery in patients [2] and animal models [3] that includes inhibition of mitochondrial pyruvate dehydrogenase complex (PDC) activation via upregulation of pyruvate dehydrogenase kinase 4 isoform (PDK4) [4].

The mitochondrion is primarily responsible for generating >90% of the cellular energy supply, in the form of adenosine triphosphate (ATP). It is versatile in its ability to do this by utilizing several different substrates, although glucose and free fatty acids make up the primary fuels. The PDC, is a multi-enzyme complex located on the inner mitochondrial membrane. It controls the irreversible conversion of pyruvate to mitochondrial acetyl-CoA, the rate limiting step in carbohydrate oxidation, and is downregulated in skeletal muscle within 2 h of the onset of *in vivo* endotoxemia in an animal model [5], and within 6–8 h of admission of patients to the intensive care unit [6]. Furthermore, several inflammatory mediators, including cytokines, have been reported to induce mitochondrial dysfunction *in vitro* via changes in the activity of mitochondrial enzyme complexes and the mitochondrial membrane potential, and consequently rates of ATP formation [[7], [8], [9], [10]]. In short, therefore, inflammation-mediated impairment of PDC and mitochondrial function play an important role in the acute dysregulation of muscle metabolism in inflammatory conditions. Moreover, mitochondria isolated the second day following surgical trauma in pigs were reported to exhibit diminished ADP stimulated respiration for pyruvate [3], although the relevance of this post-surgical response to the acute human surgical condition remains unclear.

As outlined above, we have shown that major abdominal surgery acutely resulted in marked inflammation (TNF-α and IL-6) and molecular (MAFbx, cathepsin-L, FOXO1, PDK4) and metabolic (glycogen and lactate content) dysregulation predictive of increased muscle protein degradation and impaired carbohydrate oxidation in the rectus abdominis muscle local to the site of surgical trauma [2]. However, whilst these local responses were mirrored by a similar, albeit blunted, mRNA expression response in the vastus lateralis (VL) muscle distant to the site of injury, significant changes in muscle glycogen and lactate contents and proteins regulating muscle inflammation, protein breakdown and carbohydrate oxidation were not evident in this muscle [2]. This suggests muscle energy homeostasis was not overtly compromised distant to the site of surgery, at least acutely. On the other hand, human cell culture and animal research have demonstrated that general anesthetics can profoundly impair mitochondrial function in a number of organs [[11], [12], [13], [14], [15], [16], [17]]. Furthermore, the limited human research available corroborates this. For example, skeletal muscle mitochondrial respiration was impaired in patients during general anesthesia for hip prosthesis placement or femoral fracture repair compared with the same procedures being performed under regional anesthesia [18]. The extent to which this impairment of muscle mitochondrial function during general anesthesia was attributable to surgical trauma-related inflammation, general anesthesia or the combination of both is, however, unknown.

This aim of this *post hoc* study, therefore, was to determine whether major elective abdominal surgery had any acute negative impact on PDC activity and mitochondrial function in the VL muscle distant to the site of surgical trauma.

## 2 Methods

This study comprised a *post hoc* consideration of data generated from VL muscle samples previously collected and analysed [2]. With the exception of VL muscle mRNA measurements of the cytokines interleukin-6 (IL-6) and tumour necrosis factor alpha (TNF-α) [2], all data presented in this manuscript are unpublished [i.e. muscle pyruvate dehydrogenase complex activity, citrate synthase activity and maximal mitochondrial ATP production rates (MAPR, 5 substrate mixtures) pre- and post-surgery].

Fifteen adult participants (mean age ± SEM, 49.0 ± 4.5 years, body mass index 26.0 ± 1.7 kg/m^2^, 13 males) undergoing major elective open abdominal surgery were studied, following UK national research ethics service approval and informed written consent. Seven of these patients were operated for pancreatic cancer and eight were for benign disease, such as chronic pancreatitis and incisional herniae. The protocol was registered at ClinicalTrials.gov (NCT01134809). Exclusion criteria included emergency surgery, liver surgery, chronic illness (e.g. diabetes mellitus), disseminated malignant disease, and medications known to affect glucose metabolism, statins, and chronic use of antibiotics, anti-inflammatory drugs, or immunosuppressive agents.

At the start and end of abdominal surgery, a biopsy was obtained from the VL muscle, using a percutaneous conchotome technique. The pre-surgery sample was collected 45–65 min after the start of induction of anesthesia, but prior to commencement of the operation, and the post-surgery sample was obtained from the contralateral limb at the end of the operation to avoid any impact of local tissue injury from the pre-surgery biopsy. In order to assay mitochondrial function, mitochondria were immediately isolated from a portion of muscle tissue and re-suspended in working buffers immediately after biopsy sampling (further details below). The immediacy of the procedure reflects the necessity to maintain the mitochondrial double membrane structure and its viability, which is crucial when assessing MAPR. The remaining piece of muscle was immediately frozen in liquid nitrogen.

### 2.1 Muscle IL-6 and TNF-α mRNA

Total RNA was extracted from approximately 25 mg of wet muscle using Tri Reagent (Ambion, Fisher Scientific, Loughborough, UK), and quantified spectrophotometrically at 260 nm, with RNA purity being determined as the ratio of 260/280 nm readings. Thereafter, first strand cDNA synthesis was carried out, and mRNA expression levels of IL-6 and TNF-alpha were determined using TaqMan PCR (ABI prism 7900 HT sequence detector, Applied Biosystems, Warrington, UK), as described previously [2].

### 2.2 Muscle pyruvate dehydrogenase complex activity

A portion of frozen wet muscle (5–10 mg) was used to measure PDC in its dephosphorylated (active) form (PDC_a_), as described previously [19]. Briefly, muscles were homogenized in a buffer containing NaF and dichloroacetate (DCA), and the activity of PDC in its dephosphorylated (active) form was measured as a rate of acetyl-CoA formation (mmol/min/kg wet muscle) at 37 °C. Acetyl-CoA was determined as [^14^C]citrate after condensation with [^14^C]-oxaloacetate by citrate synthase.

### 2.3 Muscle maximal mitochondrial ATP production rates

Following removal of the biopsy sample from the thigh, approximately 40 mg of muscle tissue (retained on ice) was immediately diced finely on a cooled glass plate and weighed in mg to an accuracy of two decimal places (Mettler Toledo scales, model XS105, Leicester, UK). The diced muscle was then homogenized for 3 min on ice, using a Teflon pestle homogenizer and a glass homogenization vessel (Camlab Homogeniser, model K43, Camlab Ltd., Cambridge, UK), in homogenization buffer [pH 7.2, KCl 100 mM, KH_2_PO_4_ 50 mM, Tris 50 mM, MgCl_2_ 5 mM, EDTA 1 mM, ATP 1.8 mM (all from Sigma Aldrich, Gillingham, UK)]. The crude homogenate was centrifuged at 650 *g* for 3 min, at 4 °C (Hettich Refrigerated Centrifuge, model EBA12R, DJB Labcare Ltd., Newport Pagnell, UK). The resultant supernatant was transferred to a clean test tube and centrifuged at 15,000 *g* for 3 min, at 4 °C. Following this, the supernatant was removed and discarded before resuspending the mitochondria-rich pellet in the original homogenization buffer. This was then centrifuged at 15,000 *g* for 3 min, at 4 °C. After removal of the supernatant, the pellet was resuspended in a resuspension solution (pH 7.2, human serum albumin 0.5 mg/ml, sucrose 240 mM, monopotassium phosphate 15 mM, magnesium acetate tetrahydrate 2 mM, EDTA 0.5 mM). The mitochondrial suspension was then retained on ice prior to assessing MAPR, or was frozen at −80 °C for analysis of citrate synthase activity at a later date (see below).

MAPR were determined on the mitochondrial suspension under optimal conditions utilizing a mixture of substrates (glutamate + succinate, glutamate + malate, palmitoylcarnitine + malate, pyruvate + malate, and succinate) employing a bioluminescence based technique as described previously [20], with the increase in luminescence due to the production of ATP being measured. Briefly, isolated mitochondria were introduced to a luciferase-based monitoring reagent (BioTherma, Handen, Sweden), along with ATP-free adenosine diphosphate (ADP 18 mM) and a predetermined mitochondrial substrate mixture (e.g. pyruvate + malate), and the rate of increase in luminescence due to the production of ATP was recorded. Typically, isolated muscle mitochondria were retained on ice, 12 cuvettes were employed each containing of 800 μl luciferase based ATP production monitoring reagent (lyophilised ATP monitoring reagent, BioTherma, Sweden), containing firefly luciferase, d-luciferine 0.1 g/L, l-luciferine 4 mg/L, bovine serum albumin 1 g/L, and Na_2_P_2_O_7_ 1 μM) dissolved in (sucrose 0.19 M, monopotassium phosphate 19 mM, magnesium acetate tetrahydrate 2.5 mM, EDTA 0.7 mM pH 7), 140 μl of a substrate mixture (see above), 50 μl 0.6 mM ADP (Sigma Aldrich, UK), and (added last) 10 μl of diluted mitochondrial suspension (1:100) in the same resuspension buffer. The cuvettes were then transferred to the luminometer (BioOrbit 1253 Luminometer, Turku, Finland) to quantify luminescence (mV) over time. As luminescence is directly a result of the ATP content, luminescence steadily increased as ATP was being produced by the active and intact, isolated respiring mitochondria. After 3–5 min, cuvettes were injected with 3 μl of 50 μM ATP standard, triggering a sudden increase (spike) in luminescence, which was allowed to continue for a few more minutes before the recording was stopped. The addition of the ATP standard allowed the internal conversion of the relative mitochondrial activity (mV/min) into absolute rates (μM ATP/min).

This highly sensitive method measures mitochondrial ATP production directly, and unlike classical respirometry is not affected negatively by mitochondrial uncoupling, where mitochondrial ATP production extrapolated from the measurement of oxygen consumption can be prone to overestimation. Therefore, direct measurement of mitochondrial ATP production provides a novel and valuable insight.

### 2.4 Muscle citrate synthase activity

Muscle citrate synthase activity was measured spectrophotometrically on each frozen aliquot of muscle mitochondrial suspension as described previously [21] and was used to normalize MAPR to mitochondrial content, i.e. determination of intrinsic MAPR.

### 2.5 Statistical analysis

All data are expressed as mean ± SEM. Differences between pre- and post-surgery muscle measurements were determined using the Student t-test for paired samples (for parametric data) and related samples Wilcoxon Signed Ranks Test (for non-parametric data). Differences were considered significant at p < 0.05.

## 3 Results

The mean ± SEM time elapsed between the start and the end of surgery was 223 ± 22 min (range 110–390 min).

### 3.1 Muscle IL-6 and TNF-α mRNA

Following surgery, VL muscle IL-6 and TNF-α mRNA were increased 126-fold (p < 0.05) and 5.8-fold (p < 0.05), respectively, compared with the pre-surgery expression levels [2].

### 3.2 Muscle citrate synthase activity

There was no difference in muscle citrate synthase activity from pre- to post-surgery (2.05 ± 0.23 vs 1.67 ± 0.33 nmol/min/ml mitochondrial suspension, respectively, p = 0.393), indicating mitochondrial density was no different between sampling time points.

### 3.3 Muscle pyruvate dehydrogenase complex activity

**Fig. 1:**
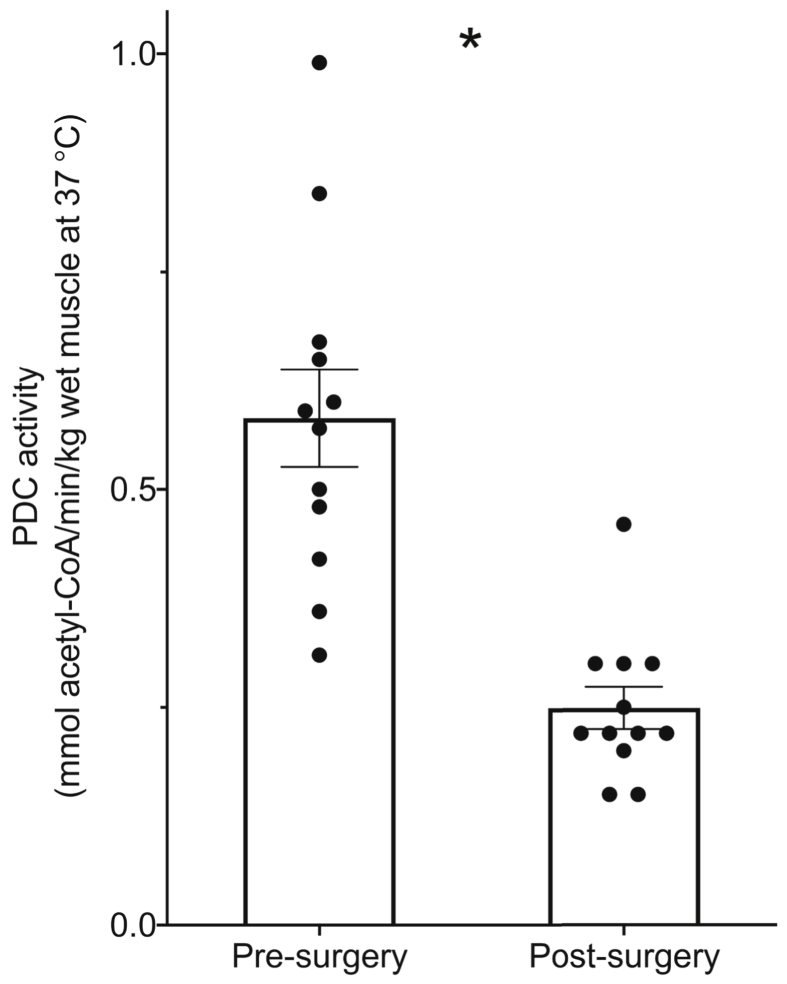
Muscle pyruvate dehydrogenase complex (PDC) activity in vastus lateralis muscle pre and post major abdominal surgery. Values represent mean ± SEM and individual values. Difference between pre and post-surgery: ∗p < 0.05.

Muscle PDC activity was reduced >50% at the end of surgery compared with pre-surgery (Fig. 1, p < 0.05).

Table 1 demonstrates there was no difference in muscle PDC activity when comparing patients with pancreatic cancer and patients with benign disease either pre- or post-surgery.

**Table 1:** Maximal mitochondrial ATP production rates and pyruvate dehydrogenase complex activity in vastus lateralis muscle pre and post major abdominal surgery in patients with pancreatic cancer (n = 7) and patients with benign disease (n = 8).

Substrate	Pyruvate dehydrogenasecomplex activity	Glutamate + succinate	Glutamate + malate	Palmitoylcarnitine + malate	Pyruvate + malate	Succinate
**Pre-surgery**						
Patients with cancer	0.51 ± 0.06	1.16 ± 0.12	0.77 ± 0.11	0.52 ± 0.06	0.43 ± 0.03	0.12 ± 0.03
Patients with benign disease	0.66 ± 0.09	0.82 ± 0.09∗∗∗	0.69 ± 0.06	0.42 ± 0.05	0.38 ± 0.06	0.19 ± 0.05
**Post-surgery**						
Patients with cancer	0.22 ± 0.02	0.76 ± 0.09	0.43 ± 0.07	0.33 ± 0.04	0.26 ± 0.03	0.12 ± 0.04
Patients with benign disease	0.28 ± 0.04	0.54 ± 0.07∗	0.41 ± 0.06	0.28 ± 0.04	0.21 ± 0.02	0.14 ± 0.04

Values represent mean ± SEM. Mitochondrial ATP production rates are μmol ATP/mmol acetyl-CoA/min (at 25 °C) and pyruvate dehydrogenase complex activity is mmol acetyl-CoA/min/kg wet muscle (at 37 °C). Significant difference between corresponding time-points between patients with pancreatic cancer and patients with benign disease: ∗p < 0.05; ∗∗∗p < 0.001.

### 3.4 Muscle maximal mitochondrial ATP production rates

**Fig. 2:**
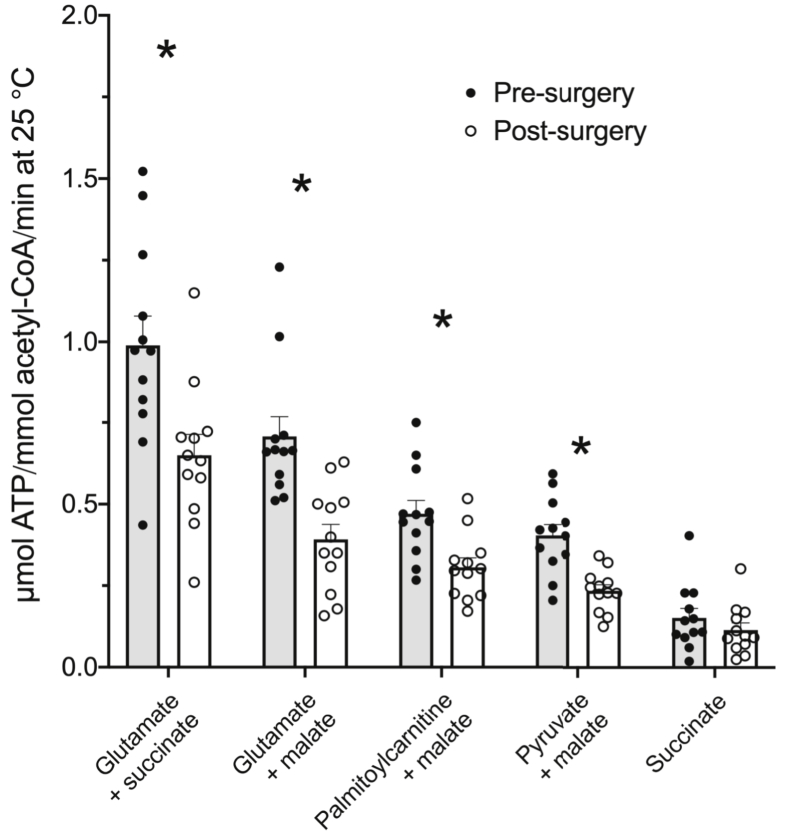
Maximal mitochondrial ATP production rates in vastus lateralis muscle pre and post major abdominal surgery. Maximal ATP production rates were determined utilizing the substrate combinations depicted on the X-axis. Values represent mean ± SEM and individual values. Differences between pre- and post-surgery: ∗p < 0.05.

Mitochondrial ATP production rates declined by ~40% from pre- to post-surgery when glutamate + succinate, glutamate + malate, palmitoyl-carnitine + malate, and pyruvate + malate were used as mitochondrial substrates (Fig. 2, all p < 0.05). There was no impact of surgery on succinate-derived mitochondrial ATP production (Fig. 2).

Table 1 illustrates that MAPR for the substrate combination of glutamate and succinate were greater in patients with cancer pre- (p < 0.001) and post-surgery (p < 0.05) when compared with those with benign disease. No other difference was found when comparing the two patient groups.

## 4 Discussion

This study aimed to assess the impact of major abdominal surgery on PDC activity and mitochondrial function in a large muscle group (VL) distant to the site of surgical trauma. The findings demonstrate clear and comprehensive declines in muscle PDC activity and mitochondrial MAPR that occurred from pre- to post-surgery, and in the absence of changes in muscle glycogen and lactate contents and proteins reflecting muscle inflammation, atrophy and impaired carbohydrate oxidation.

We have previously demonstrated that inflammation-mediated impairment of PDC plays an important role in the acute (within 2 h) dysregulation of muscle metabolism in an animal model of endotoxemia [5], and in patients within 6–8 h of admission to the intensive care unit [6]. Consistent with these observations, we [2] and others have also shown that inflammation local to the site of surgery plays an important role in the comprehensive dysregulation of muscle metabolism in surgical patients [[22], [23], [24]]. Other studies have also reported that cytokines influence skeletal muscle catabolism both directly by modulating protein synthesis and degradation and indirectly, through inhibition of the regulatory action of anabolic hormones [25] and activation of the hypothalamic-pituitary-adrenal axis [26].

The present study focused on the VL muscle distant to the site of surgical trauma, in which there was limited evidence of inflammation or changes in protein or fuel metabolism apart from mRNA changes [2]. However, with the exception of succinate, which feeds into complex II of the electron transport chain (ETC), major declines in MAPR for all substrate combinations employed that feed into complex I were observed in the same muscle sample immediately following surgery. Malate, along with pyruvate, generates oxaloacetate, and subsequently citrate via the tricarboxylic acid (TCA) cycle. Acetyl-CoA from pyruvate and palmitate derived acyl-CoA also generates citrate, whilst glutamate is utilized to generate α-ketoglutarate, another component of the TCA cycle. Collectively, these substrates produce the necessary TCA cycle intermediates for NADH production that feeds into complex I of the ETC. Furthermore, the increased availability of these intermediates shifts the dynamic equilibrium of the TCA cycle, essentially blunting succinate conversion to fumarate and the production of FADH_2_ by the TCA cycle, which diminishes the donation of electrons to complex II of the electron transport chain. Overall, therefore, the data depicted in Fig. 2 clearly suggest that the declines in MAPR observed as a result of major abdominal surgery were focused on pathways that feed electrons into complex I of the ETC. This observation is also in keeping with the reduction in PDC activity observed following surgery (Fig. 1). Furthermore, the declines in MAPR cannot be attributed to any reduction in the mitochondrial content given citrate synthase activity was unchanged from pre- to post-surgery.

In the absence of vigorous evidence of muscle inflammation, what might then explain the comprehensive changes in VL muscle PDC activity and mitochondrial MAPR that occurred from pre- to post-surgery? Changes in systemic insulin and fuel substrate availability are known to alter PDC activity and mitochondrial fuel oxidation [27,28], but neither plasma insulin or free fatty acid concentrations were found to change appreciably from pre- to post-surgery [2]. Furthermore, neither would explain the suppression of MAPR seen in the present study. More compelling is the evidence that general anesthetics and muscle relaxants have been reported to impair mitochondrial function in human cell culture [17] and animal models [29]. Furthermore, of particular relevance to the current findings halogenated anesthetics [11,[13], [14], [15]], barbiturates [12] and propofol [16,17] have been reported to inhibit oxidation of substrates entering at mitochondrial complex I level, and were included in the standard list of drugs used for anesthesia in the current study (Table 2). Animal-based drug intervention models tend to be pharmacological rather than physiological in nature and it is, therefore, of importance to note that Miro et al. [18] reported a common decrease in isolated mitochondrial respiratory capacity in skeletal muscle obtained during general anesthesia in patients undergoing hip prosthesis placement or femoral fracture repair when compared with those undergoing the same procedures under regional anesthesia. Furthermore, these events were not be explained by inhibition of ETC complex activity or uncoupling of oxidative phosphorylation [18]. Whilst in agreement with the findings of the present study, the authors [18] did not collect pre- and post-surgery muscle biopsy samples nor did they determine tissue inflammatory status, which was likely to have been marked since muscle was obtained from the quadriceps muscle group during hip or femur related surgery. Therefore, a definitive conclusion about the relative contribution of surgical trauma-related inflammation and general anesthesia to the decline in mitochondrial respiration observed was not possible. We acknowledge that because pre- and post-surgery muscle biopsies in the present study were obtained whilst patients were anesthetized we cannot be definitive about the relative contributions of surgical trauma and general anesthesia to the decline in mitochondrial function reported herein. However, muscle biopsies were obtained as soon as possible following the start of induction of anesethesia (45–65 min), and given there was no evidence of a substantive inflammatory response in the VL during surgery [2], it does seem reasonable to conclude that general anesthesia was also a contributor to the mitochondrial dysfunction observed.

**Table 2:** The standard list of drugs used for anesthesia for the patients studied.

Epidural	Intravenous	Inhalational
Bupivacaine Fentanyl	Midazolam Fentanyl Propofol Rocuronium Metaraminol (some cases) Noradrenaline (some cases, occasionally) Neostigmine (for reversal) Glycopyrrolate (for reversal) Sugammadex (occasionally, for reversal)	Desflurane or sevoflurane

In conclusion, the results of the present study demonstrate that during major elective abdominal surgery PDC activity and MAPR are acutely impaired in VL muscle distant from the site of surgical trauma. Furthermore, these events occurred under conditions where pre- to post-surgery changes in systemic insulin and free fatty acid concentrations, muscle glycogen and lactate contents and the expression of proteins reflecting muscle inflammation, atrophy and impaired carbohydrate oxidation were absent, but muscle mRNAs were differentially expressed. These findings further the understanding of the acute dysregulation of mitochondrial function in muscle distant to the site of major surgical trauma in patients, and point to the combination of general anesthesia and trauma-related inflammation as being drivers of muscle metabolic insult that warrants further investigation.

## Funding

This research was supported by the 10.13039/501100000265Medical Research Council [grant number MR/K00414X/1] and 10.13039/501100000341Arthritis Research UK [grant number 19891]. The National Institute of Health Research (NIHR) Nottingham Biomedical Research Centre and Nottingham University Hospitals Charities also supported the work. RA was supported by a Biotechnology and Biological Sciences Research Council Doctoral Training Studentship. KKV was supported by a Research Fellowship from the 10.13039/501100007743European Society for Clinical Nutrition and Metabolism.

## Role of funding bodies

The funders had no role in the study design, conduct of the study, data collection or analysis or interpretation, and writing of the paper or the decision to submit for publication. No payment has been received from any other source or agency. The corresponding author has full access to all the data in the study and has final responsibility for the decision to submit for publication.

This paper presents independent research funded by the 10.13039/501100000272National Institute for Health Research. The views expressed are those of the authors and not necessarily the views of the NHS, the NIHR or the Department of Health.

## Author contributions

RA - study design, literature search, data collection, conducting experiments, data analysis, data interpretation, writing of the manuscript and final approval.

DT-C - study design, literature search, data collection, conducting experiments, data analysis, data interpretation, writing of the manuscript and final approval.

KKV - study design, literature search, data collection, data analysis, data interpretation, writing of the manuscript and final approval.

DC - study design, conducting experiments, data analysis, data interpretation, writing of the manuscript and final approval.

DNL - study design, literature search, data interpretation, writing of the manuscript, critical review, supervision and final approval.

PLG - study design, literature search, data interpretation, writing of the manuscript, critical review, supervision and final approval.

## Conflict of interest

None of the authors has a direct conflict of interest to report.

DN Lobo has received an unrestricted research grant for unrelated work from B. Braun in the last 3 years. He has also received speakers' honoraria from B. Braun, Fresenius Kabi, Shire and Baxter Healthcare for unrelated work in the last 3 years.
